# Mutation increasing β-carotene concentrations does not adversely affect concentrations of essential mineral elements in pepper fruit

**DOI:** 10.1371/journal.pone.0172180

**Published:** 2017-02-16

**Authors:** Nasya B. Tomlekova, Philip J. White, Jacqueline A. Thompson, Emil A. Penchev, Stephan Nielen

**Affiliations:** 1 Department of Breeding, Variety Maintenance and Introduction, Maritsa Vegetable Crops Research Institute, Plovdiv, Bulgaria; 2 Department of Ecological Sciences, The James Hutton Institute, Invergowrie, Dundee, United Kingdom; 3 Department of Breeding, Dobroudja Agricultural Institute, General Toshevo, Bulgaria; 4 Joint FAO/IAEA Division, International Atomic Energy Agency, Vienna International Centre, Vienna, Austria; Huazhong University of Science and Technology, CHINA

## Abstract

Vitamin and mineral deficiencies are prevalent in human populations throughout the world. Vitamin A deficiency affects hundreds of millions of pre-school age children in low income countries. Fruits of pepper (*Capsicum annuum* L.) can be a major dietary source of precursors to Vitamin A biosynthesis, such as β-carotene. Recently, pepper breeding programs have introduced the orange-fruited (*of*) trait of the mutant variety Oranzheva kapiya, which is associated with high fruit β-carotene concentrations, to the mutant variety Albena. In this manuscript, concentrations of β-carotene and mineral elements (magnesium, phosphorus, sulphur, potassium, zinc, calcium, manganese, iron and copper) were compared in fruit from P31, a red-fruited genotype derived from the variety Albena, and M38, a genotype developed by transferring the orange-fruited mutation (*of*) into Albena. It was observed that fruit from M38 plants had greater β-carotene concentration at both commercial and botanical maturity (4.9 and 52.7 mg / kg fresh weight, respectively) than fruit from P31 plants (2.3 and 30.1 mg / kg fresh weight, respectively). The mutation producing high β-carotene concentrations in pepper fruits had no detrimental effect on the concentrations of mineral elements required for human nutrition.

## Introduction

Fruits and vegetables are an important dietary source of many of the vitamins and minerals required by humans [1–4]. Plant carotenoids, such as β-carotene, are precursors of Vitamin A biosynthesis and provide a major source of Vitamin A to human diets [5–6]. It is estimated that Vitamin A deficiency affects up to 190 million pre-school age children and 19 million pregnant women in countries with a Gross Domestic Product (GDP) < 15 000 $ *per capita* in 2005 [7]. Vitamin A deficiency is associated with increased risk of ocular disorders, degenerative diseases, respiratory, urinary and intestinal disorders, cardiovascular disease, and certain types of cancer [7–9]. Dietary intake of carotenoids can mitigate against these risks [6]. In addition, β-carotene improves the bioavailability of zinc and iron, which are also often lacking in human diets [10–11]. Thus, increasing β-carotene in food is of great importance.

Pepper fruits can often be a major dietary source of carotenoids, especially β-carotene [12–14]. There is considerable natural variation in β-carotene concentrations in pepper fruit [15–21]. In addition, induced mutations have increased β-carotene concentrations in pepper fruit [22–27]. For example, the orange-fruited variety Oranzheva kapiya, whose fruit has a high β-carotene concentration, was developed after subjecting dry seeds of a red-fruited variety, Pazardzhishka kapiya 794, to X-ray mutagenesis [22, 28]. The mutation conferring the orange-fruited (*of*) trait of Oranzheva kapiya appears to be in the 3’-terminal region of the *CrtZ* gene, which encodes an enzyme that converts β-carotene to β-cryptoxanthin [24]. Recently, this mutation was introduced into the variety Albena, which possesses the anthocyanin-free (*al*) mutation, has early and high yield, attractive fruit and better flavour [29], and a promising genotype (M38) with excellent agronomic characteristics and fruit quality has been developed [30]. The M38 genotype was registered in the working collection at the Maritsa Vegetable Crops Research Institute. The fruit from lines with orange fruit arising from the same breeding program as M38 have high β-carotene concentrations [24, 31]. However, it is important that the mutation that has resulted in high β-carotene concentrations has not affected other nutritional attributes of the fruit. Thus, the aim of this study was to check that the induced mutations producing high β-carotene concentrations in the fruits of M38 plants had no unforeseen effects on the concentrations of essential mineral elements in these fruit.

## Materials and methods

### Plant genotypes

**Fig 1:**
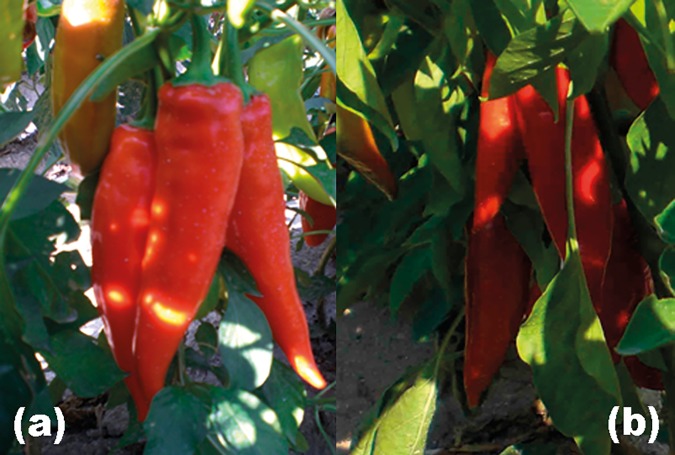
Pepper genotypes with fruit in technical and botanical maturity phase. a) M38 with orange colour of mature fruit; b) P31 with red colour of mature fruit.

The sweet pepper (*Capsicum annuum* L.) genotypes analysed in this study were P31, a red-fruited genotype derived from the variety Albena [29], and M38 (Okal38) (Fig 1), an orange-fruited genotype obtained by transferring the orange-fruited mutation (*of*) from the variety Oranzheva kapiya into Albena [28] and advancing to M_8_ isogenic lines. Both P31 and M38 are early ripening, anthocyanin-free (*al*), have high yield potential, and attractive fruit with an excellent flavour [25].

### Growth conditions and harvested portions

Ten plants of each genotype were grown in the field at the Maritsa Vegetable Crops Research Institute, Plovdiv, Bulgaria. The plants were grown in furrows following conventional practice for mid-early pepper production in this region. For mineral analyses, six ripe fruit from each plant were collected as green fruit at commercial maturity. The shape (length and diameter), thickness of pericarp, and fresh weight (FW) of each fruit was determined. Fruit were dried at 105°C for one hour then at 60°C to a constant weight to determine their dry weight (DW). For analyses of β-carotene, nineteen green fruit from P31 and nine green fruit from M38 were analysed at commercial maturity, and fifteen red fruit from P31 and ten orange fruit from M38 were analysed at botanical maturity. Fruit of each maturity x genotype were of uniform size and colour.

### Mineral analysis

Dried fruit were milled to a powder using a ball-mill. Accurately weighed subsamples of powdered fruit (approximately 50 mg DW) were digested with 3.0 mL concentrated nitric acid and 1.0 mL of 30% (v/v) hydrogen peroxide in closed vessels using a microwave digester (MARS Xpress; CEM Microwave Technology, Buckingham, UK) as described by Subramanian et al. [32]. Each digested subsample was then diluted to 50 mL with sterile MilliQ water (18.2 MΩ cm) prior to elemental analyses. The potassium (K), calcium (Ca), magnesium (Mg), phosphorus (P), sulphur (S), iron (Fe), manganese (Mn), zinc (Zn) and copper (Cu) content of digested subsamples were determined by inductively-coupled mass spectrometry (ICPMS; ELAN DRCe; PerkinElmer, Waltham, MA, USA) using argon as the carrier gas [32]. Blank digestions were performed to determine background concentrations of mineral elements, and the National Institute of Standards and Technology (NIST, Gaithersburg, MD, USA) tomato leaf standard (Reference Number 1573a) was analysed every 20 samples as an analytical control. All data were scaled to the certified composition of the tomato leaf standard (27.0 mg K / g DW, 50.5 mg Ca / g DW, 12.0 mg Mg / g DW, 2.16 mg P / g DW, 9.60 mg S / g DW, 368 μg Fe / g DW, 246 μg Mn / g DW, 30.9 μg Zn / g DW, 4.7 μg Cu / g DW). Apparent recoveries of elements from the tomato leaf standard were: K 88%, Ca 103%, Mg 129%, P 77%, S 78%, Fe 149%, Mn 122%, Zn 72%, Cu 85%.

### Beta-carotene analysis

Biochemical analyses were performed on the day on which fruit were collected. Carotenoids were extracted in duplicate from 5 g FW of green fruit or 2 g FW of red or orange fruit into acetone: ethanol: hexane (1: 1: 2) with MgCO_3_ as neutralizing agent, sonicated for 1 min, and centrifuged at 3000 rpm for 3 min, as described by Periago et al. [33]. The pellet was reextracted following the same procedure until the supernatant was colorless. The extract was dried using a rotary vacuum evaporator (Büchi Labortechnik, Flawil, Switzerland) at 30°C at 210 mbar increasing gradually to 1400 mbar. The extract was redissolved in 1 mL acetone containing 0.1% butylhydroxytoluene (BHT) as antioxidant, and then filtered through 0.45 μm Nylon membrane (EMD Millipore, Billerica, MA, USA) into dark vials. Samples were extracted and stored in the dark under nitrogen. Most of the mature fruit samples were diluted 2–3 fold before analysis of β-carotene concentration.

Beta-carotene content was determined on redissolved extracts using high performance liquid chromatography (HPLC). A 50 μl sample was injected into an Agilent Model 1200 HPLC (Agilent Technologies, Santa Clara, CA, USA), equipped with a quaternary pump and two detectors (a diode array detector set at 450 nm and a refractometer). Separation was performed for 22 min per analysis on a ZORBAX Eclipse XDB-C18 column (150 x 4.6 mm ID; Agilent Technologies) at flow rate of 0.8 ml /min. The column temperature was maintained at 20°C during the separation process. The mobile phase consisted of: A (acetonitrile and 0.1% BHT), B (acetone and 0.1% BHT), and C (water). The gradient used to separate β-carotene, targeted in the present study, and lycopene was: in 1 min 60% А and 40% С, in 10 min 35% В and 12% С, in 12 min 100% В.

Compounds were identified by their retention time and absorption spectra compared to those of known standards (CaroteNature, Ostermundigen, Switzerland). Data analysis was performed using ChemStation software (Agilent Technologies). All chemicals were HPLC grade and obtained from Merck (Darmstadt, Germany).

Six point calibration curves (n = 3 replicates) for both lycopene and β-carotene were linear in the working range of 0.04–5 μg / mL (correlation coefficients > 0.99). The limits of detection (LOD), defined as the concentration of the carotenoid resulting in a peak height three fold greater than the baseline noise, were 0.0171 μg / mL for lycopene and 0.0117 μg / mL for β-carotene. The limits of quantification (LOQ), set at 2.5 fold the LOD [34], were 0.0428 μg / mL for lycopene and 0.0293 μg / mL for β-carotene. The average short-term reproducibility (the same sample determined within a day), calculated as the coefficient of variation, was 6.0%. The average long-term reproducibility (the same sample determined 10 times over the following two days) was 7.4%. To determine the recovery of added analytes, samples were spiked with concentrations of each carotenoid from 0.07 to 0.5 μg / mL. The recoveries of the carotenoids were 97% for lycopene and 95% for β-carotene.

### Statistical analysis

Data were expressed as mean ± standard error of the mean (SE) from n determinations, unless otherwise specified. Student’s t-test was used to determine the significance of the difference between two sets of data. Statistical calculations were performed using Microsoft Office Excel (Microsoft Corporation, Redmond, WA, USA) or SPSS Version 17.0 (SPSS, Chicago, IL, USA).

## Results

Fruits from P31 and M38 plants differed in their morphological characteristics (Table 1).

**Table 1:** Morphological characteristics and ß-carotene concentrations of fruit from P31 and M38 plants. Data are expressed as mean ± standard error of the mean of n fruit.

Fruit Character		P31	M38
**Length**	(cm)	11.43 ± 0.23 (n = 60)	10.97 ± 0.21 (n = 59)
**Diameter**	(cm)	3.48 ± 0.09 (n = 60)	2.74 ± 0.07 (n = 59)
**Fresh Weight**	(g)	44.50 ± 1.50 (n = 60)	33.68 ± 1.34 (n = 59)
**Pericarp Thickness**	(mm)	3.40 ± 0.12 (n = 60)	2.98 ± 0.10 (n = 59)
**Dry Weight / Fresh Weight**	(%)	9.89 ± 0.21 (n = 60)	11.33 ± 0.23 (n = 59)
**Beta-carotene Concentration (at commercial maturity)**	(mg / kg FW)	2.3 ± 0.4 (n = 19)	4.9 ± 0.9 (n = 9)
**Beta-carotene Concentration (at botanical maturity)**	(mg / kg FW)	30.1 ± 2.7 (n = 15)	52.7 ± 4.7 (n = 10)

Fruits from P31 plants were longer (*p* = 0.142), wider (*p* < 0.001) and heavier (*p* < 0.001 for FW) than those from M38 plants and had a thicker pericarp (*p* < 0.0028; Table 1).

However, fruits from P31 and M38 plants had different dry weights (*p* = 0.651; Table 1). The β-carotene concentration of green fruit from M38 plants was greater than that of green fruit from P31 plants at commercial maturity, and the β-carotene concentration of orange fruit from M38 plants was greater than that of the red fruit from P31 plants at botanical maturity (Table 1).

At commercial maturity the average β-carotene concentration in green fruit of M38 was 4.9 mg / kg FW and that of P31 was 2.3 mg / kg FW. The average β-carotene concentration in orange fruit from M38 plants was 52.7 mg / kg FW and that in the red fruit from P31 plants was 30.1 mg / kg FW.

Fruits from P31 and M38 plants also differed in their mineral composition (Table 2).

**Table 2:** Concentrations of mineral elements in the pericarp dry matter of pepper fruit from P31 and M38 plants. Data are mean ± standard error of the mean of 60 fruit from P31 plants and 59 fruit from M38 plants.

Element		P31	M38
**Magnesium**	(mg / g DW)	1.50 ± 0.029	1.53 ± 0.021
**Phosphorus**	(mg / g DW)	3.08 ± 0.044	3.25 ± 0.043
**Sulphur**	(mg / g DW)	1.67 ± 0.040	1.97 ± 0.035
**Potassium**	(mg / g DW)	28.8 ± 0.48	27.8 ± 0.41
**Zinc**	(μg / g DW)	25.0 ± 0.67	26.6 ± 0.57
**Calcium**	(mg / g DW)	0.762 ± 0.047	0.805 ± 0.034
**Manganese**	(μg / g DW)	9.30 ± 0.240	9.40 ± 0.211
**Iron**	(μg / g DW)	41.5 ± 1.03	52.1 ± 3.07
**Copper**	(μg / g DW)	12.3 ± 0.22	11.5 ± 0.23

To avoid the vagaries of hydration, the concentrations of mineral elements in fruits from P31 and M38 plants were compared on a DW basis (cf. [11]). Although the concentrations of magnesium (Mg), potassium (K), zinc (Zn), calcium (Ca), manganese (Mn) and copper (Cu) were similar in fruit from P31 and M38 plants, the sulphur (S), phosphorus (P) and iron (Fe) concentrations in fruit from M38 plants were significantly (P < 0.01) greater than those in fruit from P31 plants.

## Discussion

Morphological characteristics are consistent with previous observations that the variety Albena had longer fruits with more pericarp than lines with orange fruit arising from the same breeding program as M38 used in this study [35]. The compared accessions have similar dry weight. The greatest difference between P31 with red fruits and M38 with orange fruits is that the β-carotene concentration of orange fruit exceeds significantly the β-carotene of red fruits. Both P31 and M38 belong to the “kapiya” type of sweet pepper that is consumed as green fruit and, because they ripen early, they are suitable for early market production. The values of β-carotene concentration in green fruit (M38 outperforms that of P31) compare favourably with green and red peppers available in the US, which contain 2.08 mg / kg FW and 16.24 mg / kg FW β-carotene, respectively [36](USDA 2012) and yellow, green and red peppers available in the UK, which contain 1.85 mg / kg FW, 2.65 mg / kg FW and 38.4 mg / kg FW β-carotene, respectively [37].

The US Recommended Dietary Allowance (RDA) for men and women is 900 and 700 μg retinol activity equivalents (RAE) / day, respectively [38]. The UK Reference Nutrient Intake (RNI) values for men and women are slightly lower, at 700 and 600 μg RAE / day, respectively [39]. Assuming that 12 μg β-carotene is equivalent to 1 μg RAE, then the US RDA for men and women could be provided by 10.8 mg β-carotene and 8.4 mg β-carotene, respectively. Thus, five to six of the orange fruit from M38 plants, or six to eight of the red fruit from P31 plants, would be sufficient to supply the US RDA.

Thus, the induced mutations producing high β-carotene concentrations in the fruits of M38 plants had no detrimental effects on the concentrations of mineral elements required for human nutrition in these fruit.

The concentrations of mineral elements found in the fruit of P31 and M38 plants grown in this study were similar to those of green and red peppers available in the US [36], and slightly lower (S, Ca, Mn), similar to (Mg, Fe) or slightly greater than (P, K, Zn, Cu) those reported for green and red peppers available in the UK [38]. Previous studies have indicated that fruit from different pepper varieties can vary significantly [40–41], but that the concentrations of most mineral elements often do not change during the transition from green to botanically mature fruit [42]. The concentrations of most mineral elements found in the fruit of P31 and M38 plants grown in this study were within the range found in previous studies (Mg, P, S, K, Zn, Fe), with the exception of Cu, which was higher than generally observed [36, 40–44].

## Conclusions

The present study demonstrates that β-carotene concentrations can be increased in pepper fruit without adverse effects on their mineral composition. This should not only improve the Vitamin A status of humans, but is also likely to increase the bioavailability of zinc and iron in the diet.

## Supporting information

S1 File Original data characterizing two mutant lines. (XLS)

## References

[1] World Health Organization / Food and Agricultural Organization of the United Nations [WHO/FAO]; Vitamin and mineral requirements in human nutrition; 2004. Second edition. Geneva, Switzerland: World Health Organization. Available from: http://apps.who.int/iris/bitstream/10665/42716/1/9241546123.pdf

[2] White PJ, Broadley MR. Biofortification of crops with seven mineral elements often lacking in human diets—iron, zinc, copper, calcium, magnesium, selenium and iodine. New Phytolog. 2009;182: 49–84. 10.1111/j.1469-8137.2008.02738.x 19192191

[3] Bates B, Lennox A, Prentice A, Bates C, Swan G. National Diet and Nutrition Survey: Headline Results from Years 1, 2 and 3 Combined 2008/09–2010/11. Retrieved 2016 Oct.; Available from: http://www.natcen.ac.uk/media/175123/national-diet-and-nutrition-survey-years-1-2-and-3.pdf

[4] Slavin JL, Lloyd B. Health benefits of fruits and vegetables. Advances in Nutrition. 2012;(3): 506–16. 22797986 10.3945/an.112.002154 PMC3649719

[5] Bohn T. Bioavailability of non-provitamin A carotenoids. Curr Res Nutr Food Sci Jour. 2008;4: 240–58. Bentham Science Publishers Ltd.

[6] Von Lintig J. Colours with functions: elucidating the biochemical and molecular basis of carotenoid metabolism. Ann Rev Nutr. 2010;30: 35–56. 20415581 10.1146/annurev-nutr-080508-141027

[7] World Health Organisation [WHO]. Global Prevalence of Vitamin A Deficiency in Populations at Risk 1995–2005. WHO Global Database on Vitamin A Deficiency. 2009; Geneva, Switzerland: World Health Organization. Available from: http://www.who.int/vmnis/database/vitamina/x/en/

[8] West KP. Vitamin A deficiency disorders in children and women. Food Nutr Bull. 2003;24: S78–S90. 17016949 10.1177/15648265030244S204

[9] Mason J, Rivers J, Helwig C. Recent trends in malnutrition in developing regions: vitamin A deficiency, anemia, iodine deficiency and child underweight. Food Nutr Bull. 2005;26: 59–162. 15810800

[10] White PJ, Broadley MR. Biofortifying crops with essential mineral elements. Trends Plant Sci. 2005a;10: 586–93. 16271501 10.1016/j.tplants.2005.10.001

[11] White PJ, Broadley MR. Historical variation in the mineral composition of edible horticultural products. J Hort Sci Biotech. 2005b;80: 660–7.

[12] O’Neill M, Carroll Y, Corridan B, Olmedilla B, Granado F, Blanco I, et al A European carotenoid database to assess carotenoid intakes and its use in a five-country comparative study. Brit J Nutr. 2001;85: 499–507. 11348565 10.1079/bjn2000284

[13] Chaiter Y, Rennert G, Fischler R, Rennert HS, Rozen G, Gruber SB, et al Dietary intake of carotenoid isomers in Israel. Int J Vit Nutr Res. 2007;77:6, 398–405. 2013 Hogrefe AG. 10.1024/0300-9831.77.6.398 18622950

[14] Biehler E, Alkerwi A, Hoffmann L, Krause E, Guillaume M, Lair ML, et al Contribution of violaxanthin, neoxanthin, phytoene and phytofluene to total carotenoid intake: Assessment in Luxembourg. J Food Compos Anal. 2011;25: 56–65. http://publicationslist.org/data/torsten-bohn/ref-32/Biehler_JFdCompAnal_2011.pdf

[15] Simonne AH, Simonne EH, Eitenmiller RR, Mills HA, Green NR. Ascorbic acid and provitamin A contents in unusually colored bell peppers (*Capsicum annuum* L.). J Food Compos Anal. 1997; 10: 299–311.

[16] Howard LR, Talcott ST, Brenes CH, Villalon B. Changes in phytochemical and antioxidant activity of selected pepper cultivars (*Capsicum* species) as influenced by maturity. J Agr Food Chem. 2000;48: 1713–20. 10820084 10.1021/jf990916t

[17] Wall MM, Waddell CA, Bosland PW. Variation in β-carotene and total carotenoid content in fruits of *Capsicum*. Hortic Sci. 2001;36: 746–9.

[18] Ha SH, Kim JB, Park JS, Lee SW, Cho KJ. A comparison of the carotenoid accumulation in *Capsicum* varieties that show different ripening colours: deletion of the capsanthin-capsorubin synthase gene is not a prerequisite for the formation of a yellow pepper. J Exp Bot. 2007;58: 3135–44. 10.1093/jxb/erm132 17728301

[19] Guzman I, Hamby S, Romero J, Bosland PW, O’Connell MA Variability of carotenoid biosynthesis in orange colored *Capsicum* spp. Plant Sci. 2010;179: 49–59. 10.1016/j.plantsci.2010.04.014 20582146 PMC2889374

[20] Rodriguez-Uribe L, Guzman I, Rajapakse W, Richins RD, O’Connell MA. Carotenoid accumulation in orange-pigmented *Capsicum annuum* fruit, regulated at multiple levels. J Exp Bot. 2012;63: 517–26. 10.1093/jxb/err302 21948863 PMC3245482

[21] Vera-Guzman AM, Chavez-Servia JL, Carrillo-Rodríguez JC, Lopez MG Phytochemical evaluation of wild and cultivated pepper (*Capsicum annuum* L. and *C*. *pubescens* Ruiz & Pav.) from Oaxaca, Mexico. Chil J Agr Res. 2011;71: 578–85.

[22] Daskalov S. Experimental mutagenesis and mutation breeding in pepper. Capsicum Newslett. 1991;10: 13–20.

[23] Tomlekova NB, Timina OO, Timin OY. Achievements and perspectives of sweet pepper breeding towards high beta-carotene. Acta Hortic. 2009a;830: 205–9.

[24] Tomlekova N, Todorova V, Petkova V, Yancheva S, Nikolova V, Panchev I, et al Creation and evaluation of induced mutants and valuable tools for pepper breeding programmes In: Shu QY editor Induced Plant Mutations in the Genomics Era, Rome, Italy: Food and Agriculture Organization of the United Nations 2009b pp. 187–90. Available from: http://www.fao.org/docrep/012/i0956e/i0956e00.pdf

[25] Tomlekova NB. Induced mutagenesis for crop improvement in Bulgaria. Plant Mut Rep. 2010;2: 4–27.

[26] Petrov V, Denev I, Draganov M, Timin O, Todorova V, Panchev I, et al Molecular characterization of advanced mutants for early detection of high beta-carotene concentrations in pepper breeding programmes. CR Acad Bulg Sci. 2013;66: 303–10.

[27] Daskalov S, Baralieva D. Oranzheva kapiya: A new cv. in sweet pepper with increased beta-carotene /provitamin A/ content. Mutat Breed Newslett. 1992;39: 9–10.

[28] Daskalov S. Albena: a new mutant cultivar in sweet pepper for early and mid-early field production. Hort. 1975;12: 22. Bulgarian.

[29] Daskalov S, Chalukova M, Baralieva D, Lukarska E. Biochemical investigations of an induced beta orange mutant in sweet pepper (Capsicum annuum L.) and developing cultivars with increased beta-carotene content. In: Proceedings of the 9th Eucarpia Meeting on Genetics and Breeding of Capsicum and Eggplant, 1995 August 21–25, Hungary, Budapest; 1995. p. 24–7.

[30] Timina OO, Timin OY, Fiodoroff SK, Tomlekova N. Inheritance of pericarp color pattern and β-carotene content in vegetable pepper. Vavilov Journal of Genetics and Breeding. 2011;15:3, 585–94. Russian.

[31] Subramanian NK, White PJ, Broadley MR, Ramsay G. The three-dimensional distribution of minerals in potato tubers. Ann Bot London. 2011;107: 681–91. 10.1093/aob/mcr009 PMC3064541 21289026

[32] Periago MJ, Rincon F, Aguera MD, Ros G. Mixture approach for optimizing lycopene extraction from tomato and tomato products. J Agr Food Chem. 2004;52: 5796–802. 15366823 10.1021/jf049345h

[33] Hulshof PM, Xu C, Van de Bovenkamp P, Muhilal West CE. Application of a validated method for the determination of provitamin A carotenoids in Indonesian foods of different maturity and origin. J Agr Food Chem. 1997;45: 1174–9.

[34] Todorova VY, Tomlekova NB. Morphological assessment of pepper mutant lines and influence of variation factors on variability of the studied characters. Acta Hortic. 2009;830: 297–303.

[35] United States Department of Agriculture, Agricultural Research Service [USDA-ARS] USDA National Nutrient Database for Standard Reference, Release 24. 2007; Beta-carotene datasheet [cited 2012 Aug 12]. Available from: http://www.ars.usda.gov/SP2UserFiles/Place/12354500/Data/SR24/nutrlist/sr24w321.pdf

[36] Holland B, Unwin ID, Buss DH. Vegetables, Herbs and Spices. Fifth Supplement to the Fourth Edition of McCance and Widdowson’s The Composition of Foods London, UK: Royal Society of Chemistry and Ministry of Agriculture, Fisheries and Food; 1991 Available from: www.fao.org/uploads/media/British_FCDB_cof_user_doc.pdf

[37] Trumbo P, Yates AA, Schlicker S, Poos M. Dietary reference intakes: vitamin A, vitamin K, arsenic, boron, chromium, copper, iodine, iron, manganese, molybdenum, nickel, silicon, vanadium, and zinc. Journal of the American Dietetic Association. 2001;101: 294–301. 10.1016/S0002-8223(01)00078-5 11269606

[38] Department of Health, UK Dietary reference values for food energy and nutrients for the United Kingdom. Report of the Panel on Dietary Reference Values of the Committee on Medical Aspects of Food Policy; 1991. Report on Health and Social Subjects 41. London, UK: HMSO. 1961974

[39] Jadczak D, Grzeszczuk M, Kosecka D. Quality characteristics and content of mineral compounds in fruit of some cultivars of sweet pepper (*Capsicum annuum* L.). J Elem. 2010;15: 509–15.

[40] Esayas K, Shimelis A, Ashebir F, Negussie R, Tilahun B, Gulelat D. Proximate composition, mineral content and antinutritional factors of some capsicum (*Capsicum annum*) varieties grown in Ethiopia. B Chem Soc Ethiopia. 2011;25: 451–4.

[41] Bernardo A, Martinez S, Alvarez M, Fernández A, Lopez M. The composition of two Spanish pepper varieties (Fresno de la vega and Benavente-los valles) in different ripening stages. J Food Quality. 2008;31: 701–16.

[42] Rubio C, Hardisson A, Martin RE, Baez A, Martin MM, Alvarez R. Mineral composition of the red and green pepper (*Capsicum annuum*) from Tenerife Island. Eur Food Res Technol. 2002;214: 501–4.

[43] Park H, Lee S, Jeong H, Cho S, Chun H, Back O, et al The nutrient composition of the herbicide-tolerant green pepper is equivalent to that of the conventional green pepper. Nutr Res. 2006;26: 546–8.

[44] European Food Information Resource Network [EuroFIR] Food Composition Databases. Retrieved 2012 Aug. 19, 2012. Available from: http://eurofir.net/eurofir_knowledge/european_databases.

